# Evaluation of a Program to Improve Linkage to and Retention in Care Among Refugees with Hepatitis B Virus Infection — Three U.S. Cities, 2006–2018

**DOI:** 10.15585/mmwr.mm6921a2

**Published:** 2020-05-29

**Authors:** Janine Young, Colleen Payton, Patricia Walker, Daniel White, Megan Brandeland, Gayathri S. Kumar, Emily S. Jentes, Ann Settgast, Malini DeSilva

**Affiliations:** ^1^Denver Health and Hospitals; University of Colorado School of Medicine, Aurora, Colorado; ^2^Department of Nursing and Public Health, Moravian College, Bethlehem, Pennsylvania; ^3^HealthPartners Institute, Minneapolis, Minnesota; ^4^University of Minnesota, Department of Internal Medicine; ^5^National Center for Emerging and Zoonotic Infectious Diseases, CDC.

An estimated 257 million persons worldwide have chronic hepatitis B virus (HBV) infection (*1*). CDC recommends HBV testing for persons from countries with intermediate to high HBV prevalence (≥2%), including newly arriving refugees (*2*). Complications of chronic HBV infection include liver cirrhosis and hepatocellular carcinoma, which develop in 15%–25% of untreated adults infected in infancy or childhood (*3*). HBV-infected patients require regular monitoring for both infection and sequelae. Several studies have evaluated initial linkage to HBV care for both refugee and nonrefugee immigrant populations (*4*–*9*), but none contained standardized definitions for either linkage to or long-term retention in care for chronic HBV–infected refugees. To assess chronic HBV care, three urban sites that perform refugee domestic medical examinations and provide primary care collaborated in a quality improvement evaluation. Sites performed chart reviews and prospective outreach to refugees with positive test results for presumed HBV infection during domestic medical examinations. Linkage to care (29%–53%), retention in care (11%–21%), and outreach efforts (22%–71% could not be located) demonstrated poor access to initial and ongoing HBV care. Retrospective outreach was low-yield. Interventions that focus on prospective outreach and addressing issues related to access to care might improve linkage to and retention in care.

Patients with a positive HBV surface antigen (HBsAg) test result during domestic medical examinations were included in the quality improvement evaluation; this result was used as a proxy for presumed chronic HBV infection. For patients not receiving optimal HBV care as defined by each clinic, trained personnel performed phone outreach using standardized phone scripts and certified medical interpreters. The script queried patients about HBV care, included HBV education, and emphasized the need for follow-up. Patients were advised to reestablish care with a primary care provider if they were living outside clinic catchment areas and not in care. Sites sent scripted messages on HBV best practices to primary care providers within their health systems if their patients were lost to follow-up or not receiving optimal HBV care.

From 2006 through 2012, a state-based public health refugee medical screening clinic in Denver, Colorado, (clinic A) performed domestic medical examinations and provided follow-up care for a proportion of persons screened. All clinic A patients with positive test results for HBsAg were referred to a gastrointestinal (GI) specialist for ongoing management. Chart reviews and telephone outreach for patients with positive test results for HBsAg were conducted during 2016–2018 to determine whether patients were up to date with laboratory testing. Persons who were not up to date were offered follow-up appointments; an online search database was used to find current telephone numbers for those who could not be contacted. A nurse updated problem lists in patient charts with chronic hepatitis B diagnoses.

Clinic B, in St. Paul, Minnesota, performed domestic medical examinations and provided follow-up primary and ongoing HBV care for some patients, although patients might also have received ongoing hepatitis B care through GI specialists. Patients with domestic medical examinations performed during 2008–2017 who were aged ≥18 years at the start of the quality improvement project and had received medical care within the health system during the previous 3 years were included. During 2017, chart reviews were performed to determine whether patients were up to date with laboratory testing and liver ultrasound and had at least one appointment with a GI specialist. Initial linkage to HBV care was not evaluated at clinic B because the standard of care was to obtain alanine aminotransferase (ALT) levels and HBsAg testing for all patients and reflex laboratories (i.e., HBeAg, hepatitis B e-antibody [HBeAb], hepatitis B core antibody [HBcAb], and HBV DNA) for patients with positive test results for HBsAg; hepatitis B education was provided to patients with positive test results for HBsAg at the second visit.

Clinic C, in Philadelphia, Pennsylvania, conducted domestic medical examinations and provided follow-up care. Patients with domestic medical examinations performed during 2007–2018 were included in the analysis. Refugees with positive test results for HBsAg were followed by a primary care provider, a GI specialist, or an infectious disease specialist for ongoing hepatitis B care. Chart reviews were performed to determine whether patients were up to date with laboratory tests and appointments. Telephone outreach to patients not receiving optimal hepatitis B care was conducted from 2017 through 2018.

All sites received an Institutional Review Board waiver based on quality improvement designation. A CDC human subjects advisor determined that this project did not meet the definition of research under 45 CFR 46.102(d).*

## Laboratory Results

**Clinic A.** Laboratory test results indicating initial linkage to care for clinic A included HBV DNA, ALT, HBeAg, and HBeAb; those indicating retention in care included HBV DNA and ALT (Table 1). A total of 306 refugees had positive test results for HbsAg, and among these refugees, 204 were included in evaluations by clinic A; 29% (60) had initial linkage, 12% (24) were retained in care, and 84% (172) were not receiving optimal HBV care (Table 2). Despite telephone outreach efforts, 71% of patients were lost to follow-up, and one patient was confirmed to have died from hepatocellular carcinoma.

**TABLE 1:** Hepatitis B care definitions used by three clinics caring for refugees with hepatitis B virus infection — Denver, Colorado (Clinic A); St. Paul, Minnesota (Clinic B); and Philadelphia, Pennsylvania (Clinic C), 2006–2018

Definition	Clinic A	Clinic B	Clinic C
**Linkage to hepatitis B care**	Seen by GI specialist within 12 months of domestic medical examination for hepatitis B, with HBV DNA, ALT, HBeAg, HBeAb laboratory testing completed	Not evaluated	Seen by primary or specialty care (GI or ID specialist) within 12 months of domestic medical examination for hepatitis B, with HBV DNA, ALT, HBeAg, HBeAb laboratory testing completed
**Retained in hepatitis B care**	One or more primary care or GI specialist visits after initial linkage to hepatitis B care in which HBV infection was addressed within the past 12 months, including ALT and HBV DNA	Laboratory tests within previous 3–6 months: ALT, HBV DNA, and alpha-fetoprotein	One or more primary or specialty care visits after initial linkage to hepatitis B care in which HBV infection was addressed within the past 12 months, including ALT and HBV DNA
		Liver ultrasound within previous 6–12 months	
		GI specialist appointment at any time	
**Not receiving optimal hepatitis B care**	No primary or GI specialist visit in which hepatitis B was addressed within the past 12 months, including ALT and HBV DNA	Overdue for laboratory tests or liver ultrasound and no previous GI specialist appointment	No primary or specialty visit in which hepatitis B was addressed within the past 12 months, including ALT and HBV DNA

**Abbreviations:** ALT = alanine aminotransferase; GI = gastrointestinal; HBeAb = hepatitis B e-antibody; HBeAg = hepatitis B e-antigen; HBV = hepatitis B virus; ID = infectious diseases.

**TABLE 2:** Refugee demographics and hepatitis B care quality improvement results from three clinics — Denver, Colorado (Clinic A); St. Paul, Minnesota (Clinic B); and Philadelphia, Pennsylvania (Clinic C), 2006–2018

Characteristic	No. (%)		
	Clinic A	Clinic B	Clinic C
Refugees screened for hepatitis B during domestic medical exam*	5,520 (100)	5,229 (100)	1,676 (100)
**Refugees with positive HBsAg**	306 (6)	310 (6)	53 (3)
**Refugees included in quality improvement^†^**	204 (4)	137 (3)	53 (3)
Median age, yrs (interquartile range)	31 (24–42)	34 (27–44)	29 (25–40)
Female	77 (37)	47 (34)	16 (30)
**Birth country**			
Bhutan	13 (6)	3 (2)	0 (0)
Burma	101 (50)	85 (62)	27 (51)
Democratic Republic of the Congo	6 (3)	3 (2)	6 (11)
Ethiopia	7(3)	7 (5)	0 (0)
Eritrea	9 (4)	1 (1)	0 (0)
Somalia	28 (14)	28 (20)	0 (0)
Thailand	0 (0)	7 (5)	0 (0)
Other	40 (20)	3 (2)	20 (38)
**Hepatitis B care outcomes^§^**	204 (100)	137 (100)	53 (100)
Linked to hepatitis B care	60 (29)	N/A	28 (53)
Retained in hepatitis B care	24 (12)	29 (21)	6 (11)
Not receiving optimal hepatitis B care	172 (84)	108 (79)	47 (89)
Cleared hepatitis B virus infection	7 (3)	—^¶^	—^¶^
Death from hepatocellular carcinoma**	1 (<1)	—^¶^	—^¶^
**Outreach, no. (%)^††^**	167	108	42
Could not be located	119 (71)	30 (28)	29 (69)
Receiving medical care within the health system but lost to follow-up for hepatitis B; inbox message sent to primary care provider	4 (2)	15 (14)	0 (0)
UTD laboratory and ultrasound, but no GI specialist appointment	N/A	16 (15)	N/A
GI specialist following, but laboratory and ultrasound outdated	N/A	8 (7)	N/A
Receiving hepatitis B care with outside provider	1 (<1)	16 (15)	6 (14)
Declined follow-up, no insurance	N/A	1 (1)	N/A
Scheduled appointment at clinic	N/A	22 (20)	N/A
Not in hepatitis B care	37 (22)	N/A	4 (10)
Moved, hepatitis B education letter sent	6 (4)	N/A	3 (7)

**Abbreviations:** GI = gastrointestinal; HBsAg = hepatitis B surface antigen; N/A = not available; UTD = up to date.

* Clinic A: 2006–2012; clinic B: 2008–2017; clinic C: 2007–2018.

**^†^** 102 patients excluded from Denver chart review because these patients were referred to other primary care clinics for ongoing care; charts not available. 173 patients excluded from clinic B quality improvement because patient had not been seen within the health system in the 3 years before start of quality improvement project or patient was aged <18 years at time of project start.

^§^ Clinic A: 2016–2018; clinic B: 2017–2018; clinic C: 2007–2018.

^¶^ Data not collected by clinics B and C.

** In Colorado, hepatitis B-related deaths were confirmed by matching cases to Colorado vital records.

**^††^** Outreach by patient navigators to refugees not receiving optimal hepatitis B care.

**Clinic B.** Laboratory results indicating retention in care for refugees who received domestic medical examinations at clinic B included HBV DNA, ALT, and alpha-fetoprotein. (Table 1). Among 137 of 310 refugees with positive test results for HBsAg who were included in the quality improvement follow-up, 21% (29) were retained in hepatitis B care (Table 2). Among the 79% (108) not receiving optimal hepatitis B care, 15% (16) were up to date on laboratory monitoring and ultrasound but had not been seen by a GI specialist, 20% (22) agreed to schedule appointments at clinic B to reestablish care, and 15% (16) reported receiving care for their HBV infection outside clinic B. Overall, 28% (30) of those not receiving optimal hepatitis B care could not be contacted by telephone, 7% (eight) were being followed by a GI specialist but were not up to date on laboratory testing and imaging, and 1% (one) declined follow-up because they lacked health insurance.

**Clinic C.** Laboratory tests required for initial linkage to care included HBV DNA, ALT, HBeAg, and HBeAb; those required for retention in care included HBV DNA and ALT (Table 1). Among 53 (3%) refugees with positive test results for HBsAg, 53% were initially linked to hepatitis B care, 11% were retained in care, and 47 (89%) were not receiving optimal hepatitis B care (Table 2). Outreach was attempted for 42 of 47 patients not receiving optimal care. Among 42 patients not receiving optimal hepatitis B care for whom telephone outreach had been attempted, 69% could not be located, 10% were not in HBV care, 14% were in care with an outside provider, and 7% had moved outside the jurisdiction.

## Discussion

Patients with chronic HBV infection require lifelong monitoring to prevent progression to end stage liver disease and hepatocellular carcinoma. Although it is recommended that those with positive test results for HBV receive counseling and additional evaluation to determine treatment eligibility, there are no mechanisms in place to ensure that this takes place. Despite recommended HBV screening practices during domestic refugee medical examinations, significant barriers remain for long-term management of HBV infection in refugee populations. A low percentage of refugee patients who received a diagnosis of HBV infection at domestic medical examinations attended initial hepatitis B-specific appointments (clinics A and C), and retention in care was low at all three sites, ranging from 11% to 21%. These results are similar to those of another recent study, which included mostly Asian immigrants living in the United States and found that management of chronic HBV infection was poor (*10*). Although not specifically evaluated, it is hypothesized that insufficient HBV counseling at the time of diagnosis, complicated by difficulties navigating the U.S. health system because of patients’ limited English proficiency, transportation challenges, access to insurance coverage, and other competing priorities, including need for work and income, likely affect both initial and long-term follow-up. In this investigation, retrospective outreach to refugees with hepatitis B infection was labor-intensive and low-yield in improving follow-up. Implementation of standard linkage and retention definitions would be useful in future investigations to systematically assess intervention effectiveness across multiple sites. In addition, creation of hepatitis B patient registries to provide active monitoring of patients in real time might allow for prospective outreach with the goal of improving follow-up.

The findings in this report are subject to at least four limitations. First, site-specific definitions were used to assess initial linkage to and retention in care, so numbers might be under- or overestimated. Second, patient populations varied between sites; and available community supports, education level, and cultural differences in perception of U.S. health care, HBV infection, and understanding of preventive medicine might have affected linkage and retention. Third, sites only had access to internal electronic medical records; some refugees might have been receiving care through other health systems. Finally, given that an initial positive HBsAg result was used as a proxy for presumed chronic HBV infection, the number of patients with chronic HBV infection might have been overestimated.

Identification and management of hepatitis B infection in persons from countries with a high prevalence of infection, including refugees, is important to protecting their health and preventing transmission to others; refugees are at risk for not being linked to and retained in hepatitis B care. Future efforts should focus on identification of barriers and facilitators that contribute to linkage to and retention in hepatitis B care with the goal of developing interventions to improve timely outreach and long-term follow-up.

**Summary**

**What is already known about this topic?**

CDC recommends testing of all newly arriving refugees for hepatitis B virus (HBV) infection.

**What is added by this report?**

After diagnosis of HBV infection at three U.S. refugee screening sites, rates of linkage to specialist care were low at two sites (29% and 53%) and rates of retention in care ranged from 11% to 21%. Coordinated retrospective outreach to refugees with HBV infection was labor-intensive and low-yield.

**What are the implications for public health practice?**

Implementation and evaluation of interventions to improve linkage to and retention in hepatitis B care for refugees, including comprehensive, standardized counseling at the time of diagnosis and at all follow-up visits, removing barriers to care, and real-time monitoring patient follow-up, are needed to improve disease management and prevent transmission.

## Footnotes

* https://www.hhs.gov/ohrp/sites/default/files/revised-common-rule-reg-text-unofficial-2018-requirements.pdf.

## References

[1] World Health Organization. Global hepatitis report, 2017. Geneva, Switzerland: World Health Organization; 2017. https://www.who.int/hepatitis/publications/global-hepatitis-report2017/en/

[2] CDC. Screening for viral hepatitis during the domestic medical examination of newly arrived refugees. Atlanta, GA: US Department of Health and Human Services, CDC; 2019. https://www.cdc.gov/immigrantrefugeehealth/guidelines/domestic/hepatitis-screening-guidelines.html

[3] Fattovich G, Bortolotti F, Donato F. Natural history of chronic hepatitis B: special emphasis on disease progression and prognostic factors. J Hepatol 2008;48:335–52. 10.1016/j.jhep.2007.11.011 18096267

[4] Stanford J, Biba A, Khubchandani J, Webb F, Rathore MH. Community-engaged strategies to promote hepatitis B testing and linkage to care in immigrants of Florida. J Epidemiol Glob Health 2016;6:277–84. 10.1016/j.jegh.2016.06.003 27373603 PMC7320466

[5] Walters J, Sullivan A. Early identification and linkage to care of foreign-born people with chronic hepatitis B virus infection, Multnomah County, Oregon, 2012–2014. Public Health Rep 2016;131(Suppl 2):105–11. 10.1177/00333549161310S216 27168669 PMC4853336

[6] Harris AM, Schoenbachler BT, Ramirez G, Vellozzi C, Beckett GA. Testing and linking foreign-born people with chronic hepatitis B virus infection to care at nine U.S. programs, 2012–2014. Public Health Rep 2016;131(Suppl 2):S20–8. 10.1177/00333549161310S204 27168657 PMC4853324

[7] Mitruka K, Pezzi C, Baack B, Evaluation of hepatitis B virus screening, vaccination, and linkage to care among newly arrived refugees in four states, 2009–2011. J Immigr Minor Health 2019;21:39–46. 10.1007/s10903-018-0705-x 29417356 PMC6434685

[8] Beckett GA, Ramirez G, Vanderhoff A, . Early identification and linkage to care of persons with chronic hepatitis B virus infection—three U.S. sites, 2012–2014. MMWR Morb Mortal Wkly Rep 2014;63:399–401. 24807238 PMC5779400

[9] Linde AC, Sweet KA, Nelson K, Mamo B, Chute SM. Impact of the hepatitis testing and linkage to care (HepTLC) initiative on linkage to care for Minnesota refugees with hepatitis B, 2012–2014. Public Health Rep 2016;131(Suppl 2):112–8. 10.1177/00333549161310S217 27168670 PMC4853337

[10] Nguyen VH, Le AK, Trinh HN, Poor adherence to guidelines for treatment of chronic hepatitis B virus infection at primary care and referral practices. Clin Gastroenterol Hepatol 2019;17:957–67. 10.1016/j.cgh.2018.10.012 30326298

