# *Erratum*: Vol. 69, No. 21

**DOI:** 10.15585/mmwr.mm6927a6

**Published:** 2020-07-10

In the report “Evaluation of a Program to Improve Linkage to and Retention in Care Among Refugees with Hepatitis B Virus Infection — Three U.S. Cities, 2006–2018,” on page 650, **Katherine Yun, Children's Hospital of Philadelphia** should have been listed in the Acknowledgments section.

